# How the French Give Castor Oil to Children

**Published:** 1885-09

**Authors:** 


### How the French give Castor Oil to Children.

A French method of administering castor oil to children is to pour the oil into a pan over a moderate fire, break an egg into it and stir up; when it is done, flavor with a little salt or sugar, or currant jelly.—*Med. Record.*

